# Real-world experience of angiotensin receptor neprilysin inhibitor on the glucose-lowering effect

**DOI:** 10.1038/s41598-022-13366-z

**Published:** 2022-06-11

**Authors:** Heungjo Kim, Gyunam Park, Jongsung Hahn, Jaewon Oh, Min Jung Chang

**Affiliations:** 1 Department of Pharmacy and Yonsei Institute of Pharmaceutical Sciences, Yonsei University, Incheon, Republic of Korea; 2 Department of Pharmaceutical Medicine and Regulatory Science, Yonsei University, Incheon, Republic of Korea; 3 Cardiology Division, Department of Internal Medicine, Severance Cardiovascular Hospital, Cardiovascular Research Institute, Yonsei University College of Medicine, Seoul, Republic of Korea; 4 KIURI Research Center, Ajou University, Suwon, Republic of Korea; 5 Department of Industrial Pharmaceutical Science, College of Pharmacy, Yonsei University, Incheon, Republic of Korea

**Keywords:** Cardiology, Diseases, Medical research

## Abstract

We investigated the effect of angiotensin receptor neprilysin inhibitor (ARNI) on glycemic control in Korean patients. This retrospective cohort study was conducted at a single tertiary hospital. We compared the HbA_1c_ level reduction between the ARNI and angiotensin-converting enzyme inhibitors (ACEIs) or angiotensin II receptor blockers (ARBs) in chronic heart failure patients with diabetes. We also examined whether the target HbA_1c_ level was reached and the time to start insulin between the two groups. Over the study period, ARNI did not significantly lower the HbA_1c_ level after adjusting confounding factors compared to ACEIs or ARBs. However, as a result of a simple comparison using Mann–Whitney U test, ARNI group showed significant decrease in HbA_1c_ at 6, 12, and 24 months compared to ACEIs or ARBs group (*p* = 0.003, 0.009, and 0.026, respectively). The initiation of insulin was delayed in the ARNI group, but this difference was not significant based on the result of hazard ratio, but cumulative incidence was significantly lower in the ARNI group. In the real world, the blood glucose-control effects of ARNI were not superior to those of ACEIs or ARBs. However, long-term studies are needed as ARNI use increases to obtain more statistically significant results.

## Introduction

Sacubitril/valsartan is the first drug to be approved as an angiotensin receptor-neprilysin inhibitor (ARNI)^1^. This drug was approved by the US Food and Drug Administration (FDA) in 2015 to treat chronic heart failure with reduced ejection fraction (HFrEF)^2^. According to the 2017 American College of Cardiology/American Heart Association Task Force on Clinical Practice Guidelines and the Heart Failure Society of America (ACC/AHA/HFSA), angiotensin-converting enzyme inhibitors (ACEIs), angiotensin receptor blockers (ARBs), or ARNIs are recommended for patients with chronic HFrEF to reduce morbidity and mortality as class I treatments^3^. In randomized controlled trials (RCTs) that compared sacubitril/valsartan with enalapril in symptomatic HFrEF patients who did not respond to an appropriate dose of either ACEI or ARB, ARNI was found to reduce cardiovascular death or heart failure hospitalization by 20%^4^.

In patients with diabetes mellitus (DM), the risk of heart failure is over two-fold higher than that in patients without DM^5^. While 10%–15% of the general population has diabetes, a recent study suggests that about 44% of patients hospitalized for heart failure have DM^6^. Moreover, Erqou et al.^7^ showed a linear association between HbA_1c_ and the risk of heart failure among American patients with diabetes, and Echouffo-Tcheugui et al.^8^ suggested that DM is independently associated with a greater risk of death and rehospitalization compared to that in non-diabetes patients with heart failure (HF).

Sacubitril has been reported to increase insulin sensitivity in some studies^9–11^. One recent post-hoc analysis from the PARADIGM-HF trial included 3778 patients with known diabetes or HbA_1c_ ≥ 6.5%, who were randomly assigned sacubitril/valsartan or enalapril treatment^12^. The results of this trial indicated that patients who received sacubitril/valsartan showed a greater reduction in HbA_1c_ levels than those receiving enalapril (overall reduction 0.14%). These data suggest that sacubitril/valsartan may be effective for glycemic control in patients with HF and diabetes.

However, no retrospective cohort study in the real world has been reported till date. Although retrospective studies are more susceptible to recall bias or information bias compared to RCTs, they have the advantage of better reflecting the real situation. Therefore, we compared the glucose-lowering effects of ARNI versus those of ACEIs or ARBs in patients with both DM and HF in Korea.

## Methods

### Study design and participants

This cohort study was retrospectively conducted at a single tertiary level hospital in Seoul, Korea, from January 1, 2017 to May 31, 2020. The study was approved by the Institutional Review Board (IRB No. 4-2021-0168) of the Yonsei University Health System. Since this is a retrospective cohort study, the informed consents were waived. In addition, this study was performed in accordance with the Declaration of Helsinki and approved by an appropriate ethics committee. The inclusion criteria for this study were patients diagnosed with heart failure and type 2 diabetes (ICD-10 I50.X, E11.X, E13.X), taking ‘ACEI’ or ‘ARB’ or ‘ARNI’ for at least 180 days, and aged over 19 years old. Patients who had less than 80% compliance with medication and for whom medical data were incomplete were excluded from the study.

We collected the following data for each patient: sex, birth date, age at initiation of medication, first hospital visit date, drug prescription (antidiabetic drugs, antihypertensive drugs), medical history (concomitant disease), HbA_1c_, estimated glomerular filtration rate (eGFR), and serum creatinine concentration (SCr). The eGFR was calculated using the Modified Diet in Renal Disease (MDRD) equation^13^. HbA_1c_ concentrations were measured using the BioRad D-10 Hemoglobin A_1c_ Program (Bio-Rad Laboratories Inc. Hercules, California) as the percentage determination of HbA_1c_ levels using ion-exchange high-performance liquid chromatography^14^. HbA_1c_ concentrations at 6, 12, 18, and 24 months were collected for each patient based on the medical record reviews.

### Outcomes

The primary outcome was changes in the HbA_1c_ levels at 6, 12, 18, and 24 months from the baseline. The secondary outcomes were the difference between the two drug groups in reaching the HbA_1c_ target level (less than 6.5%) and the difference of the initiation of insulin and time to initiation of insulin. Subgroup analysis based on ejection fraction was additionally performed. We also analyzed groups ARNI, ACEI, and ARB by dividing them into two groups.

### Statistical analysis

Baseline characteristics between the treatment groups were compared using the Pearson chi-square test for categorical data (e.g., sex, previous history of DM, other medical history, medications), and Student’s *t*-test (parametric method) or Mann–Whitney U test (nonparametric method) for continuous data (for e.g., age, HbA_1c_, SCr, eGFR). For each time point, the HbA_1c_ change was examined using a stepwise regression analysis. Every variable of the patient characteristics was considered when performing the regression analysis. Overall HbA_1c_ changes were assessed using the linear mixed model (LMM) method. The difference in HbA_1c_ between the baseline and each time point was analyzed using Mann–Whitney U test. Whether the target HbA_1c_ level was reached or not was analyzed using the generalized estimating equation (GEE). The proportion of patients starting insulin use and time to initiation of insulin were analyzed using the Cox proportional hazards model and Kaplan–Meier estimates. Statistical analyses were performed using SAS 9.4, (SAS Institute Inc., SAS Campus Drive, Cary, North Carolina 27513, USA. All rights reserved.) or R version 4.1.1 (The R Foundation for Statistical Computing, Vienna, Austria).

## Results

**Figure 1:**
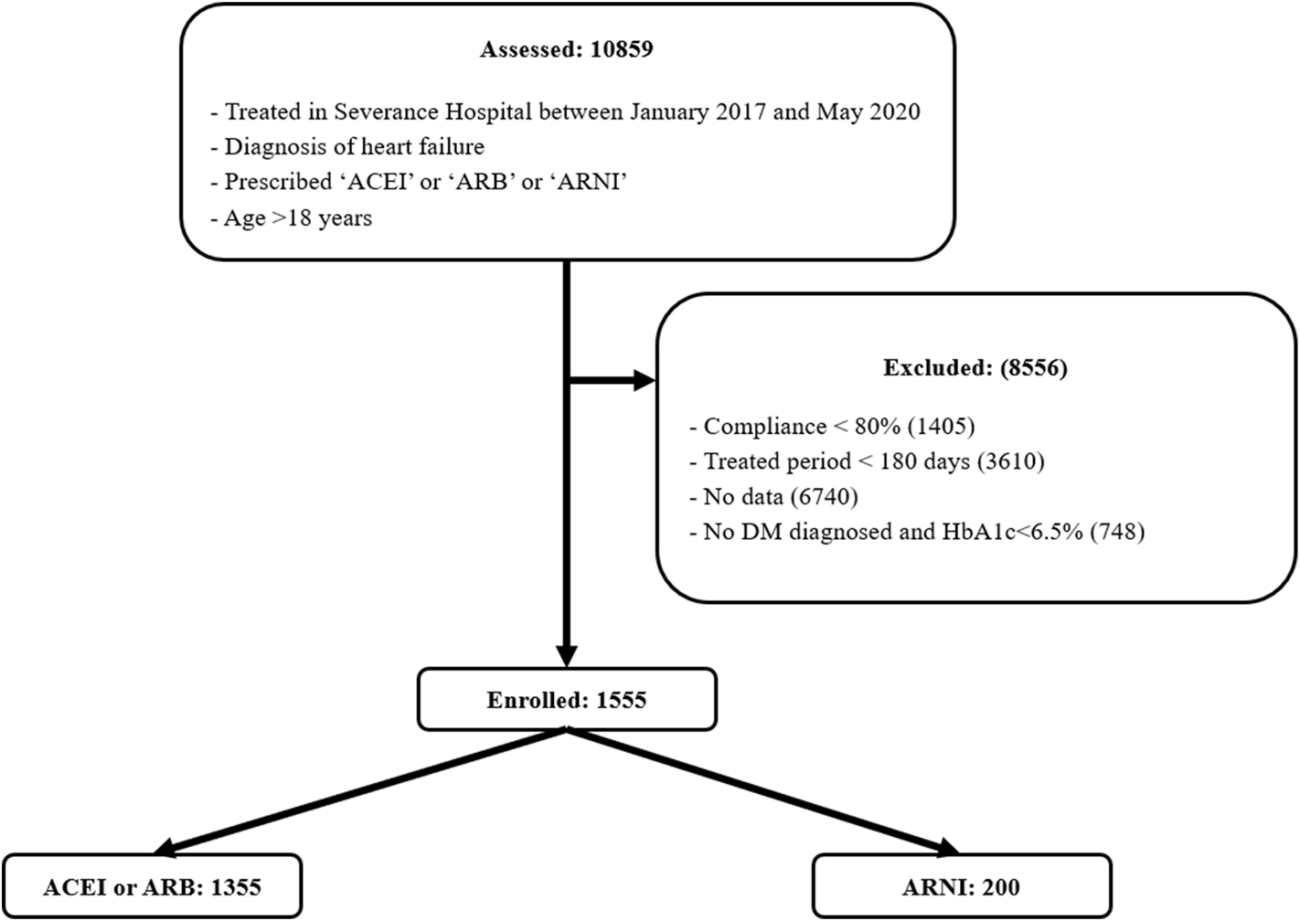
Flowchart of patient inclusion and exclusion criteria.

Of 10,859 HF patients over 18 years of age who were taking ACEIs, ARBs, or ARNI, 1555 (14%) patients were finally enrolled, as subjects with diabetes based on their medical history or a screening HbA_1c_ concentration ≥ 6.5% (Fig. 1).

The characteristics of the enrolled patients are shown in Table 1. The mean age of patients taking ACEIs or ARBs was 71 years (IQR, 62.0–78.0) and that of the patients taking ARNI was 70 years (IQR, 61.5–77.0). The proportion of male patients was higher in ARNI group than that of ACEI or ARB group (68.5% vs. 59.3%, *p* = 0.0126). There was no significant difference in the HbA_1c_ baseline level and duration of diabetes between the ARNI group and ACEI or ARB group. The ejection fraction was significantly higher in the ACEI or ARB group compared with the ARNI group (53.5% vs. 33.9%, *p* < 0.001). The median concentration of serum creatinine in the group using ARNI was 1.1 mg/dL (IQR 0.91–1.52), which was higher (*p* < 0.001) than that of the group using ACEI or ARB (0.96 mg/dL, IQR 0.78–1.26). There were several concomitant diseases whose incidence differed between the two groups, including hypertension and dyslipidemia. In addition, there was a statistically significant difference between the use of sulfonylurea, thiazolidinedione, SGLT2 inhibitors, GLP-1 receptor agonists, statins, calcium channel blockers, β-blockers, and diuretics.

**Table 1:** Baseline characteristics of patients with diabetes (overall and in the treatment groups).

	ARNI (*n* = 200)	ACEI or ARB (*n* = 1355)	*p*-value
Age (years)	70.0 (61.5–77.0)	71.0 (62.0–78.0)	0.0673
Sex			0.0126
Male	137 (68.5%)	803 (59.3%)	
Female	63 (31.5%)	552 (40.7%)	
HbA_1c_ (%)	7.00 (6.60–7.85)	6.90 (6.50–7.70)	0.1624
Ejection fraction (%)	33.9 (25–40)	53.5 (42–67)	< 0.0001
Duration of DM (days)	1125 (805–1560)	1173 (574.5–1550.5)	0.0672
Creatinine (mg/dL)	1.10 (0.91–1.52)	0.96 (0.78–1.26)	< 0.0001
eGFR (mL/min/1.73 m^2^)	61.85 (44.11–79.55)	71.45 (51.73–88.64)	0.0001
Concomitant disease			
Hypertension	142 (71.0%)	1132 (83.5%)	< 0.0001
Dyslipidemia	104 (52.0%)	909 (67.1%)	< 0.0001
Myocardial infarction	26 (13.0%)	155 (11.4%)	0.5205
Stroke	12 (6.0%)	93 (7.3%)	0.6496
Atrial fibrillation	70 (35.0%)	309 (22.8%)	0.0002
Treatment			
Metformin	118 (59.0%)	739 (54.5%)	0.2364
Sulfonylurea	73 (36.5%)	395 (29.2%)	0.0344
Thiazolidinedione	3 (1.50%)	136 (10.0%)	< 0.0001
Meglitinide or α-glucosidase inhibitors	4 (2.0%)	54 (4.0%)	0.1666
Insulin	59 (29.5%)	367 (27.1%)	0.4747
SGLT2 inhibitors	46 (23.0%)	91 (6.7%)	< 0.0001
GLP-1 receptor agonists	8 (4.0%)	1 (0.1%)	< 0.0001
Dipeptidyl peptidase 4 inhibitors	108 (54.0%)	638 (47.1%)	0.0677
Statins	169 (84.5%)	1030 (76.0%)	0.0077
Calcium channel blockers	37 (18.5%)	581 (42.9%)	< 0.0001
β-blockers	182 (91.0%)	930 (68.6%)	< 0.0001
Diuretics	189 (94.5%)	700 (51.7%)	< 0.0001

**Figure 2:**
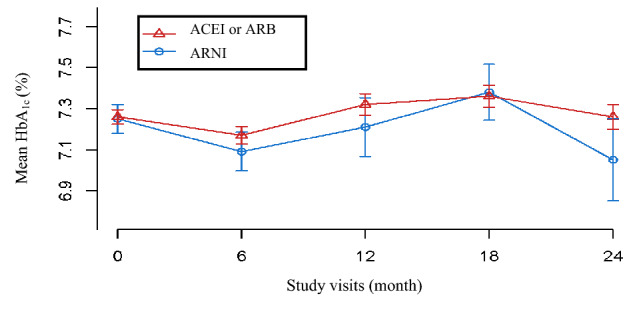
Changes in the mean HbA_1c_ levels and standard error of the means by treatment group at the baseline, 6-month, 12-month, 18-month, and 24-month visits.

Changes in the mean HbA_1c_ level at 6-month, 12-month, 18-month, and 24-month from the baseline are presented in Table 2 and Figure 2. HbA_1c_ reduction was assessed through stepwise linear regression and adjusted for factors found to be potentially predictive of HbA_1c_ reduction. During the first 6 months of the follow-up, the HbA_1c_ level decreased by 0.09% in the ACEI or ARB group and by 0.16% in the ARNI group (between-group difference, 0.13%; 95% CI −0.31–0.05; *p* = 0.1608, compared with the baseline); these statistically insignificant differences were appeared over all points. Over the full duration of follow-up, the change in HbA_1c_ level was not significantly different between patients receiving ARNI with those receiving ACEI or ARB (overall increment, 0.005; 95% CI −0.009–0.019; *p* = 0.511) (Table 2). Difference in HbA_1c_ between baseline and each time point for both groups are presented in Figure S1. A simple comparison using Mann-Whitney U test, ARNI group showed significant decrease in HbA_1c_ at 6, 12, and 24 months compared to ACEIs or ARB group (*p* = 0.003, 0.009, and 0.026, respectively). As a result, the decrease in HbA_1c_ was significantly greater in the ARNI group than in the ACEI or ARB group at all time points except 18 months.

**Table 2:** HbA_1c_ concentrations (%) in the subjects from the treatment groups over the four time points.

	ARNI (*n* = 200)	ACEI or ARB (*n* = 1355)	Adjusted values	
			Differences (95% CI)	*p*-value
Baseline^ǂ^	7.25 (1.00)	7.26 (1.31)	–	–
6 months^ǂ^	7.09 (1.12)	7.17 (1.24)	−0.13 (−0.31 to 0.05)	0.1608
12 months^ǂ^	7.21 (1.33)	7.32 (1.39)	−0.20 (−0.45 to 0.05)	0.1231
18 months^ǂ^	7.38 (0.96)	7.36 (1.30)	0.06 (−0.28 to 0.39)	0.7388
24 months^ǂ^	7.05 (1.04)	7.26 (1.37)	−0.15 (−0.60 to 0.29)	0.4948
Overall*	–	–	0.005 (−0.009 to 0.019)	0.5107

Data are expressed as the mean (SD). Number of patients with measurements of HbA_1c_ at the baseline for ‘ACEI or ARB’ and ‘ARNI’ = (1355 and 200), 6 months = (829 and 141), 12 months = (696 and 86), 18 months = (577 and 49), and 24-months = (505 and 27), respectively.

Adjusted variable: stroke, insulin, calcium channel blocker (CCB), age, eGFR.

ǂStepwise linear regression analysis. *Linear mixed model method.

As the secondary outcome, there was no significant difference between the two groups with regards to reaching the HbA_1c_ target level over time (*p* = 0.1113).

**Figure 3:**
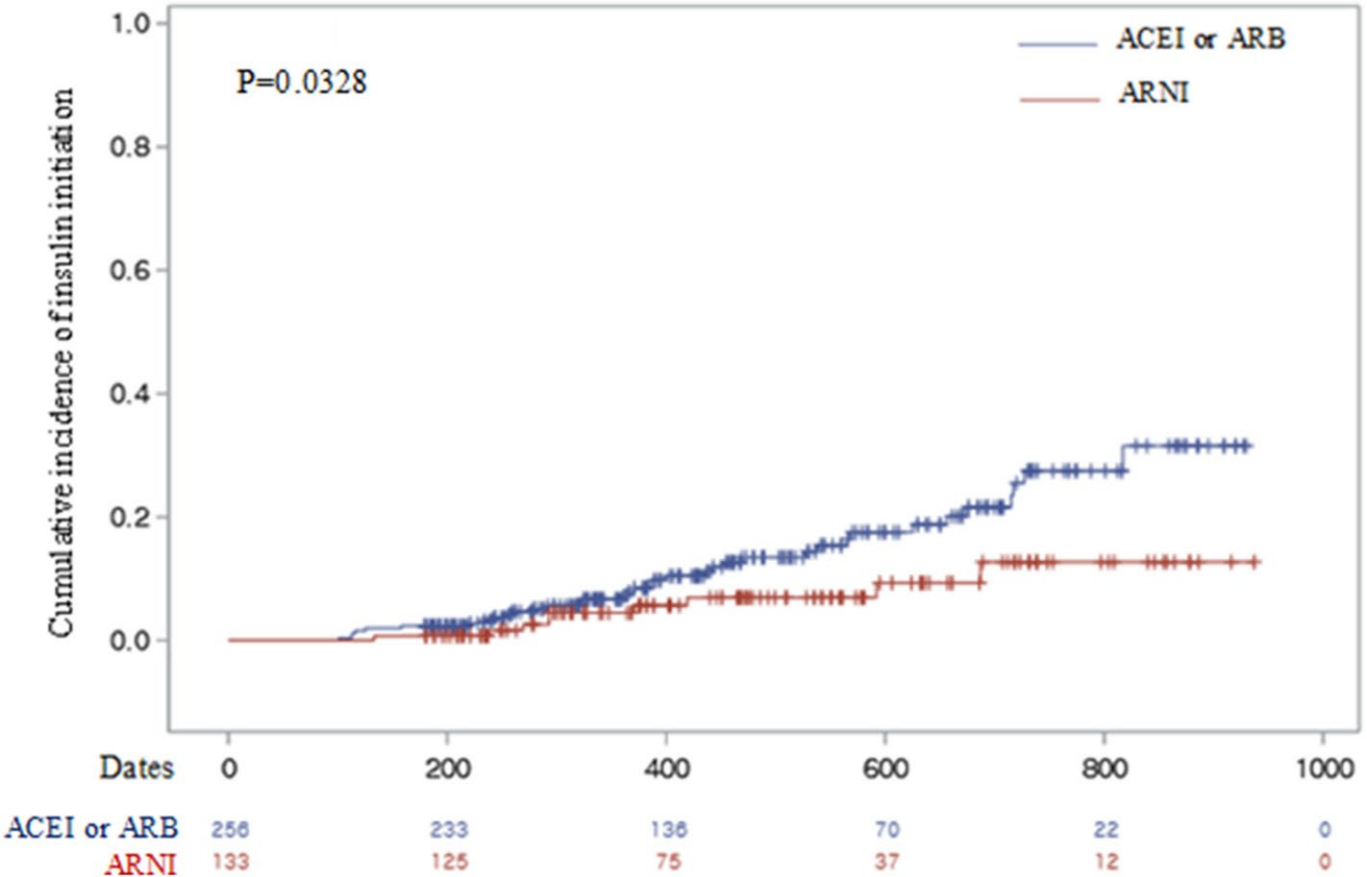
Kaplan–Meier curve showing the time to insulin initiation in patients from the ARNI and ACEI or ARB groups who were not treated with insulin previously.

Among patients with diabetes who were insulin-naive at the baseline, 36 (14%) patients in the ACEI or ARB group, and 9 (7%) in the ARNI group were initiated on insulin therapy (HR, 0.52; 95% CI 0.23–1.12; *p* = 0.094; Table 3). The Kaplan–Meier curve showing the incidence of insulin initiation in the ARNI group and ACEI or ARB group is shown in Figure 3 and it was significantly lower in the ARNI group (*p *= 0.0328).

**Table 3:** The initiation of insulin therapy in patients with diabetes, not receiving insulin at the baseline.

	ARNI (*n* = 133)	ACEI or ARB (*n* = 256)	*p*-value
Overall^ǂ^	9 (7%)	36 (14%)	–
Hazard ratio^ǂ^	0.52 (0.23–1.12)	Reference	0.094
Incidence rate per 100 person-years^ǂ^	3.15 (1.98–4.31)	6.06 (3.94–8.17)	–

ǂData are expressed as the number of subjects (%) and ratio (95% CI).

Adjusted variable: creatinine, age, SGLT2 inhibitors.

The subgroup analysis of HbA_1c_ changes in patients with an ejection fraction less than 40% are shown in figure S2. There were no statistically significant differences between two groups except for the 12-month time point. As a result of comparing the reduction of HbA_1c_ among the three drugs, there was no statistical difference between ARB and ACEI. However, ARNI group was significantly better than ACEI group or ARB group at all time points except 18 months. (Table S1).

## Discussion

According to the 2017 ACC/AHA/HFSA, ARNIs (sacubitril/valsartan) have been recommended for patients with chronic HF to reduce morbidity and mortality in a stable state, rather than ACEIs or ARBs^3^. In this study, we investigated the additional effects of ARNI on blood glucose control, compared with those of ACEIs or ARBs. We compared the HbA_1c_ level reduction, therapeutic difference in reaching the HbA_1c_ target, and the time to start insulin. Over the study period, ARNI did not significantly lower the HbA_1c_ level compared to ACEIs or ARB after adjusting confounding factors (age, stroke, insulin use, calcium channel blocker use, estimated glomerular filtration rate). However, a simple comparison ARNI group showed significant decrease in HbA_1c_ at 6, 12, and 24 months compared to ACEIs or ARB group (*p* = 0.003, 0.009, and 0.026, respectively). The initiation of insulin was delayed in the ARNI group, but this difference was not significant based on the result of HR, but cumulative incidence of insulin initiation was significantly lower in the ARNI group. To our knowledge, this is the first retrospective cohort study to reflect an actual clinical setting and to compare the effects of ARNI with those of ACEIs or ARBs on blood glucose control.

There was a statistically significant difference in HbA_1c_ reduction at 12 months between ARNI group and ACEI or ARB group regardless of EF level (Figure S1 and S2). This is a clinically meaningful result, even though it did not adjust confounding factors. The glucose-lowering effects of neprilysin inhibition occur via the modulation of the degradation of multiple peptides with glucoregulatory properties such as GLP-1, bradykinin, atrial natriuretic peptide (ANP), and B–type natriuretic peptide (BNP)^9^. If the activity of neprilysin is inhibited, the plasma concentration of these peptides is increased, which results in a glucose-lowering effect. Another pharmacological mechanism of neprilysin inhibition is the increase in glucose-stimulated insulin secretion (GSIS)^15^. It is difficult for ARNIs to show dramatic glycemic control. However, ARNIs can provide additional glucose-lowering effects in patients with heart failure, whose blood glucose levels are at the upper borderline despite high adherence to antidiabetic drugs. Controlling blood glucose levels in patients with heart failure is important because diabetes is independently associated with a greater risk of death and rehospitalization^8^.

However, there was a fluctuation in the HbA_1c_ level, decreasing for 6 months, and then rising to its highest value at 18 months, and then falling for 24 months (Fig. 2). This might reflect a decrease in the overall compliance after 6 months of treatment. In the long-term treatment of diabetes, it is necessary to establish medication adherence to increase the likelihood of treatment success. It has been reported that at least 45% of patients with type 2 diabetes fail to achieve adequate glycemic control (HbA_1c_ < 7%)^16^. One of the major contributing factors for this is poor adherence to medication^17^. In this study, patients with a medication adherence greater than 80% were included. However, it was difficult to determine the actual medication adherence because this criterion was calculated based on the number of days of prescription. In addition, patients with heart failure have many concomitant diseases; therefore, there may be problems with polypharmacy, which in turn, may lead to poor medication adherence.

In this study, many patients were taking various drugs including diabetes medications (thiazolidinedione, SGLT2 inhibitors and GLP-1 agonist) which may have influenced the blood sugar changes. However, during stepwise linear regression analysis, diabetes drugs were not selected as confounding variables. The confounding variables were stroke, insulin, calcium channel blocker (CCB), age, and eGFR.

Because EF of patients using ACEI or ARB tended to be higher than those of ARNI, we considered subgroup analysis based on EF of less than 40%. Similar to the results of all patients, there was significant differences in HbA_1c_ reduction between two groups at 12 months (*p* = 0.006, Figure S2). Therefore, it means that EF was not a factor that made the glucose lowering effect of ARNI greater.

In previous studies, ARB and ACEI were already reported to reduce blood glucose level^18,19^. When comparing the reduction of HbA_1c_ among the three drugs of ARNI, there was no statistical difference between ARB and ACEI. However, ARNI group was significantly better than ACEI group or ARB group at all time points except 18 months, which suggests at least ARNI has the definitive effect on lowering HbA_1c._

In the cumulative incidence of insulin initiation, there was significant difference between two groups. However, the ARNI group started insulin at only about one-third of that in the ACEI or ARB group without statistical significance although the hazard ratio was only 0.52.

Although we found a possible glycemic control effect of ARNI, there are some limitations to this study. ARNI is a new drug approved by the US FDA in 2015; thus, the clinical experience is relatively short. Therefore, the relatively small number of patients could have led to statistical insignificance. Additionally, incomplete data, such as the absence of periodic follow-up regarding the HbA_1c_ level, may also have contributed to the limitations. Since this study is a single-center retrospective study, a large-scale retrospective study of several institutions in the future needs to be conducted by matching these baseline characteristics through propensity score. However, our study could better reflect the actual clinical setting compared to post hoc study performed with RCT^12^.

Nevertheless, this study presents the possibility of a glucose-lowering effect and delay of DM progression when ARNIs are used in heart failure patients with type 2 DM. In the future, further studies are necessary which define the glucose level-lowering effect of ARNI with a large number of patients.

## Conclusion

In the real world, we found that there was a trend toward a decrease in blood glucose over time and delayed initiation of insulin in ARNI group compared to ACEI or ARB group. ARNI can be more beneficial than ACEI or ARB in patients with diabetes. In the future, long-term studies are needed as ARNI use increases to obtain more statistically significant results.

## Supplementary Information

Supplementary Information 1.

Supplementary Information 2.

Supplementary Information 3.

Supplementary Information 4.

## Data Availability

Data available on request due to privacy/ethical restrictions.
