# Supplementary material for: Real-world experience of angiotensin receptor neprilysin inhibitor on the glucose-lowering effect
Source: Sci Rep. 2022 Jun 11;12:9703. doi: 10.1038/s41598-022-13366-z (PMC9188559; doi:10.1038/s41598-022-13366-z)
Supplement: Supplementary file 2 — Supplementary Information 2. [file 41598_2022_13366_MOESM2_ESM.docx]

P=0.107

P=0.117

P=0.653

P=0.006

Difference (HbA_1c_)

Appendix supplement figure 2. Differences in baseline and HbA_1c_ at 6-months, 12-months, 18-months, and 24-months according to medications in patients with EF less than 40%.
