# Supplementary material for: Real-world experience of angiotensin receptor neprilysin inhibitor on the glucose-lowering effect
Source: Sci Rep. 2022 Jun 11;12:9703. doi: 10.1038/s41598-022-13366-z (PMC9188559; doi:10.1038/s41598-022-13366-z)
Supplement: Supplementary file 3 — Supplementary Information 3. [file 41598_2022_13366_MOESM3_ESM.docx]

|  | ARNI | ACEI | ARB |
| --- | --- | --- | --- |
| 6 months (%) ^ǂ^ | -0.23 | -0.092 | -0.203 |
| 12 months (%) ^ǂ^ | 0.23 | 0.016 | -0.043 |
| 18 months (%) ^ǂ^ | 0.01 | 0.073 | -0.023 |
| 24 months (%) ^ǂ^ | -0.25 | -0.079 | -0.068 |
| ^ǂ^ HbA_1c_ difference between baseline and each time point. | | | |

Table S1. Results of comparing the reduction of HbA_1c_ (%) among the three drugs at 6, 12, 18, and 24 months.

|  | ARNI vs. ACEI | ARNI vs. ARB | ACEI vs. ARB |
| --- | --- | --- | --- |
| 6 months * | 0.039 | 0.04 | 0.7779 |
| 12 months * | 0.028 | 0.025 | 0.386 |
| 18 months * | 0.64 | 0.34 | 0.275 |
| 24 months * | 0.042 | 0.02 | 0.749 |
| ^*^ P-value between each drug. | | | |

Table S1-1. Results of statistical analysis of blood glucose difference between drugs.
